# Increased mitochondrial Ca^2+^ contributes to health decline with age and Duchene muscular dystrophy in *C. elegans*

**DOI:** 10.1096/fj.202201489RR

**Published:** 2023-03-19

**Authors:** Atsushi Higashitani, Mika Teranishi, Yui Nakagawa, Yukou Itoh, Surabhi Sudevan, Nathaniel J. Szewczyk, Yukihiko Kubota, Takaaki Abe, Takeshi Kobayashi

**Affiliations:** ^1^ Graduate School of Life Sciences Tohoku University Sendai Japan; ^2^ Medical Research Council (MRC) Versus Arthritis, Centre for Musculoskeletal Ageing Research, Royal Derby Hospital University of Nottingham Derby UK; ^3^ Ohio Musculoskeletal and Neurologic Institute, Heritage College of Osteopathic Medicine Ohio University Athens Ohio USA; ^4^ College of Life Sciences Ritsumeikan University Kusatsu Japan; ^5^ Division of Medical Science Tohoku University Graduate School of Biomedical Engineering Sendai Japan; ^6^ Department of Clinical Biology and Hormonal Regulation Tohoku University Graduate School of Medicine Sendai Japan; ^7^ Graduate School of Medicine Nagoya University Nagoya Japan

**Keywords:** aging, calcium, dystrophy, MCU, mitophagy, sarcopenia

## Abstract

Sarcopenia is a geriatric syndrome characterized by an age‐related decline in skeletal muscle mass and strength. Here, we show that suppression of mitochondrial calcium uniporter (MCU)‐mediated Ca^2+^ influx into mitochondria in the body wall muscles of the nematode *Caenorhabditis elegans* improved the sarcopenic phenotypes, blunting movement and mitochondrial structural and functional decline with age. We found that normally aged muscle cells exhibited elevated resting mitochondrial Ca^2+^ levels and increased mitophagy to eliminate damaged mitochondria. Similar to aging muscle, we found that suppressing MCU function in muscular dystrophy improved movement via reducing elevated resting mitochondrial Ca^2+^ levels. Taken together, our results reveal that elevated resting mitochondrial Ca^2+^ levels contribute to muscle decline with age and muscular dystrophy. Further, modulation of MCU activity may act as a potential pharmacological target in various conditions involving muscle loss.

## Graphical abstract

In *C. elegans* body wall muscle, resting [Ca^2+^]_mito_ levels increased with age, and high‐intensity dots of mitochondria‐targeted red fluorescent Ca^2+^ indicators were frequently separated from highly connected mitochondria. These processes proceeded by mitophagy, which accelerated mitochondrial fragmentation and volume loss. [Ca^2+^]_mito_ levels were also elevated, leading to progressive muscle wasting in a model of muscular dystrophy. Inhibition of Ca^2+^ influx into mitochondria via the mitochondrial calcium uniporter alleviated mitochondrial dysfunction and improved muscle health in both aging and dystrophic models.

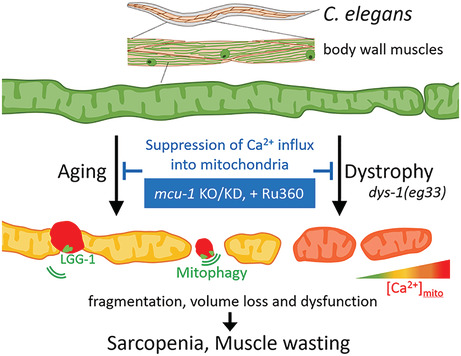

**Abbreviations**
[Ca^2+^]_cyto_
: cytosolic Ca^2+^
[Ca^2+^]_mito_
: mitochondrial Ca^2+^
DMD: Duchenne muscular dystrophy
FWHM: full‐width half‐maximum
MCU: mitochondrial calcium uniporter
mtGECO: mito‐LAR‐GECO1.2
mtGFP: mitochondrial‐targeted GFP
NGM: Nematode growth medium
nucGFP: nuclear‐targeted GFP
OCR: oxygen‐consumption rate
RFU: relative fluorescence unit
ROS: reactive oxygen species
RyR: ryanodine receptor
SERCA: sarco/endoplasmic reticulum Ca^2+^‐ATPase
UPR^mt^
: mitochondrial unfolding protein response
WT: wild type

## 1 INTRODUCTION

Sarcopenia is an age‐related skeletal muscle disorder characterized by the accelerated loss of muscle mass and strength.^1^, ^2^, ^3^ The skeletal muscles of aged mice and older people both display mitochondria with altered features including decreased volume, irregular morphology, and decreased functional activities.^4^, ^5^, ^6^ Mitochondrial Ca^2+^ has been shown to regulate crucial mitochondrial functions such as energy production, reactive oxygen species (ROS) production, and the initiation of cell death.^7^ Recently, it has been reported that aged mice display significantly increased levels of resting mitochondrial Ca^2+^ in skeletal muscle fibers,^4^ indicating that dysregulated mitochondrial Ca^2+^ homeostasis could be involved in sarcopenia. Mitochondrial calcium uniporter (MCU) has been identified to be the primary channel responsible for mitochondrial Ca^2+^ uptake across the inner mitochondrial membrane.^8^, ^9^ Therefore, suppression of MCU function was expected to prevent undesirable accumulation of Ca^2+^ in mitochondria and to ameliorate sarcopenia. However, Rizzuto's group has demonstrated that MCU silencing in rodent skeletal muscle causes muscle atrophy, and that overexpression of MCU in the hindlimb of mice, increasing mitochondrial Ca^2+^ uptake, causes muscle hypertrophy and provides a protective effect against denervation‐induced atrophy.^10^ Conversely, mutations in an MCU regulator, MICU1, which increase resting mitochondrial Ca^2+^ levels, caused neuromuscular disorders with cognitive decline, muscle weakness, and an extrapyramidal motor disorder.^11^ Discrepancies in these findings indicate multiple roles of mitochondrial Ca^2+^ on skeletal muscle homeostasis. Namely, mitochondrial Ca^2+^ may have distinct effects on various processes of skeletal muscle aging. Therefore, to study the role of mitochondrial Ca^2+^ in sarcopenia, it is crucial to verify its role in a simple experimental system.

The body wall muscle in *Caenorhabditis elegans* has a structure similar to vertebrate skeletal muscle containing sarcomeres. Also similar to mammalian muscle, *C. elegans* muscle displays structural and functional declines with age.^12^, ^13^ *C. elegans* sarcomeres and mitochondria are located in the monolayer within the cell and can be easily observed alive under a microscope. Declines in mitochondrial network structure, increased fragmentation, and reduced mitochondrial volume occur earlier than sarcomere decline and correlate more strongly with a reduction in movement, maximum velocity, and life span.^14^, ^15^, ^16^ In addition, several molecular systems such as the dystrophin complex and mitophagy, which is controlled by PINK and PERKIN, are conserved in *C. elegans*.^17^, ^18^, ^19^ Furthermore muscle deterioration can be examined without the influence of muscle regeneration since *C. elegans* has no muscle stem cells. Therefore, the *C. elegans* body wall muscle is a simple model useful to study (primary) sarcopenia and other inherited muscular diseases.

In this study, we examined the role of mitochondrial Ca^2+^ homeostasis in the context of aging and sarcopenia using *C. elegans*. Initially, like past studies, we observed aberrant changes in mitochondria in the body wall muscle of aged worms. We next confirmed elevated levels in resting mitochondrial Ca^2+^ with age. Either pharmacologic or genetic inhibition of MCU function was sufficient to prevent increases in mitochondrial Ca^2+^ and improve sarcopenic phenotypes. In addition, we found that Duchene muscular dystrophy (DMD) worms also exhibit abnormally high cytosolic and mitochondrial Ca^2+^ levels and that MCU inhibition was similarly sufficient to prevent increases in mitochondrial Ca^2+^ and improve health. The results of this study indicate that altered mitochondrial Ca^2+^ homeostasis is associated with muscle aging and dystrophy in *C. elegans*. These findings raise the possibility that mitochondrial Ca^2+^ homeostasis is associated with mammalian muscle aging and dystrophy and that it may be a potential therapeutic target in them.

## 2 MATERIALS AND METHODS

### 2.1 *C. elegans* strains and culture conditions

We followed standard procedures for *C. elegans* strain maintenance.^20^ All strains were cultured on nematode growth medium (NGM) plates with OP50 as a food source at 20°C. The worms were synchronized by egg laying for 3 h. The following strains were used in this study: N2 wild‐type, CZ19982: *mcu‐1(ju1154)*, BZ33: *dsy‐1(eg33)*, SD1347: *ccIs4251* [*Pmyo‐3::GFP‐LacZ(NLS) + Pmyo‐3::mitochondrial GFP*], ATU2301: *goeIs3* [*Pmyo‐3::GCaMP3.35::unc‐54‐3′utr, unc‐119(+)*] *aceIs1* [*Pmyo‐3::mitochondrial LAR‐GECO + Pmyo2::RFP*], ATU2302: *mcu‐1(ju1154) goeIs3 [Pmyo‐3::GCaMP3.35::unc‐54‐3′utr, unc‐119(+)] aceIs1 [Pmyo‐3::mitochondrial LAR‐GECO + Pmyo2::RFP]*, ATU2305: *dys‐1(eg33) goeIs3* [*Pmyo‐3::GCaMP3.35::unc‐54‐3′utr, unc‐119(+)*] *aceIs1* [*Pmyo‐3::mitochondrial LAR‐GECO + Pmyo2::RFP*], ATU3301: *ccIs4251 [Pmyo‐3::nucGFP‐LacZ + Pmyo‐3::mitochondrial GFP*] *aceIs1* [*Pmyo‐3::mitochondrial LAR‐GECO+ Pmyo2::RFP*], ATU3302: *pdr‐1(gk448) ccIs4251 [Pmyo‐3::nucGFP‐LacZ + Pmyo‐3::mitochondrial GFP*] *aceIs1* [*Pmyo‐3::mitochondrial LAR‐GECO+ Pmyo2::RFP*], ATU3304: *mcu‐1(ju1154) ccIs4251* [*Pmyo‐3::nucGFP‐LacZ + Pmyo‐3::mitochondrial GFP*] *aceIs1* [*Pmyo‐3::mitochondrial LAR‐GECO+ Pmyo2::RFP*], ATU3305: *dys‐1(eg33) ccIs4251* [*Pmyo‐3::nucGFP‐LacZ + Pmyo‐3::mitochondrial GFP*] *aceIs1* [*Pmyo‐3::mitochondrial LAR‐GECO+ Pmyo2::RFP*], ATU3309: *dys‐1(eg33) mcu‐1(ju1154) ccIs4251* [*Pmyo‐3::nucGFP‐LacZ + Pmyo‐3::mitochondrial GFP*] *aceIs1* [*Pmyo‐3::mitochondrial LAR‐GECO+ Pmyo2::RFP*], ATU4301: *aceIs1* [*Pmyo‐3::mitochondrial LAR‐GECO + Pmyo2::RFP*], ATU4303: *adIs2122* [*Plgg‐1::lgg‐1::GFP*] *aceIs1* [*Pmyo‐3::mitochondrial LAR‐GECO + Pmyo2::RFP*], and ATU4304: *pwIs50* [*Plmp‐1::lmp‐1::GFP*] *aceIs1* [*Pmyo‐3::mitochondrial LAR‐GECO + Pmyo2::RFP*].

### 2.2 Imaging of mitochondrial morphology, mitochondrial Ca^2+^, cytosolic Ca^2+^, and nuclear morphology

Mitochondrial morphology and mitochondrial Ca^2+^ in body wall muscle cells were observed by assaying expression of the transgenes *ccIs4251 [Pmyo‐3::nucGFP‐LacZ + Pmyo‐3::mitochondrial GFP]*,^21^ *goeIs3 [myo‐3p::SL1::GCamP3.35::SL2::unc54 3′UTR + unc‐119(+)]*,^22^ and *aceIs1 [Pmyo‐3::mitochondrial LAR‐GECO + Pmyo2::RFP]*, respectively. Synchronized worms were mounted on a microscope slide with a 6.5‐mm square, 20‐μm deep well made with a water‐repellent coating (Matsunami Glass Ind., Ltd., Osaka, Japan) with a 100 mM NaN_3_ solution (Z‐stack imaging) or 2.5% polystyrene microspheres (0.10 μm, Polysciences Inc., Warringston, PA, USA) (time‐lapse confocal imaging). Z‐stack images and time‐lapse confocal images of GFP and mtGECO fluorescence were observed by an FV10i confocal laser‐scanning microscope (Olympus Co., Tokyo, Japan). Time‐lapse confocal images of cytosolic GCaMP and mtGECO fluorescence in body wall muscle cells were acquired at room temperature (20–22°C) on a CSU‐W1 spinning disk scanner (single camera split‐view model, Yokogawa Electric Co., Ltd., Tokyo, Japan) on an Eclipse Ti2‐E inverted microscope (Nikon, Tokyo, Japan) with a CFI Apo TIRF 60x N.A. 1.49 objective (Nikon). Worms were simultaneously illuminated by two laser lines at 488 nm (Sapphire 488, Coherent Inc., Santa Clara, CA, USA) and 561 nm (OBIS, Coherent). Emission fluorescence of GCaMP and mtGECO was divided by a dichroic mirror (561LP, IDEX Corp, Lake Forest, IL, USA) and projected onto adjacent halves of an EMCCD camera (iXon Life 888; Andor Technology, Belfast, UK). Images were acquired every 1 s for calcium imaging and analyzed using of NIS‐Elements AR software (Nikon).

Mitochondria were grouped into categories by morphology according to Regmi et al.^14^ as follows: “tubular,” images indicating a majority of long interconnected mitochondrial networks; “intermediate,” images indicating a combination of interconnected mitochondrial networks along with some smaller fragmented mitochondria; “fragmented,” images indicating a majority of short mitochondria; and “very fragmented,” images indicating sparse small round mitochondria. The morphological categories of mtGECO were grouped into categories as follows: “matched with GFP,” images that match with mitochondrial morphology were classified as matched with GFP; “small dots,” images indicating more than 10 dots per cell smaller than 1 μm were classified as small dots; “large dots,” images indicating more than 10 dots per cell larger than 1 μm were classified as large dots; and “lines,” images indicating more than 5 linear signals per cell were classified as lines.

### 2.3 Measurement of mitochondrial Ca^2+^ and cytosolic Ca^2+^ levels

Mitochondrial Ca^2+^ and cytosolic Ca^2+^ in body wall muscle cells were observed by assaying expression of the transgenes *aceIs1 [Pmyo‐3::mitochondrial LAR‐GECO + Pmyo2::RFP]* and *goeIs3 [Pmyo‐3::GCaMP3.35::unc‐54‐3′utr, unc‐119(+)]*,^22^ respectively. The fluorescent signals of mtGECO in a constant area and cytosolic GCaMP in the whole body were imaged by an FV10i confocal laser‐scanning microscope (Olympus). The Ca^2+^ concentration in muscle mitochondria ([Ca^2+^]_mito_) was calculated using the following equation^23^:

$${\left[{\mathrm{Ca}}^{2+}\right]}_{\text{mito}}=K_{d}\cdot \left(R-R_{\min}\right)/\left(R_{\max}-R\right),$$

where K_d_ (12 μM) indicates the dissociation constant between Ca^2+^ and the LAR‐GECO probe,^24^ and R indicates the ratio of fluorescence intensity of mtGECO to that of mtGFP in a constant area. Muscle mitochondria at each age were exteriorized in the incision of worms cut by a blade and calibrated in the presence of 10 mM EGTA (R_min_) or by the addition of 5 μM Ionomycin in 1 mM CaCl_2_ (R_max_) (Figure S1A,B).

### 2.4 Measurement of life span

To measure life span, the living and dead worms were counted every day. The survival of worms was determined by touch‐provoked movement. A total of 100–120 worms were placed on four replicate plates, with 25–30 worms per plate. The worms were transferred to a fresh plate every 2–3 days.

### 2.5 Measurement of mean velocity and locomotion activity

Synchronized worms were transferred to NGM plates with no bacteria. Movement was recorded by using stereomicroscopy (SMZ18; Nikon), a device camera (DP74; Olympus), and imaging software (cellSens Standard 2.2; Olympus). The distance moved every 5 s was measured, and the average of four points was calculated. To determine locomotion activity, we calculated the average thrashing rate after tapping in liquid culture for 10 s.^17^, ^25^

### 2.6 Measurement of the OCR

Synchronized worms were washed three times with M9 buffer, and five worms per well were transferred to a 96‐well, black wall, clear bottom plate containing 45 μL of M9 buffer. Five microliters of reagent from a MitoXpress Xtra Oxygen Consumption Assay kit (Agilent Technologies, Santa Clara, CA, USA) was added per well and mixed. The wells were covered with mineral Oil, and fluorescence was detected with a microplate reader (Spark 10 M; Tecan, Männedorf, Switzerland). The rate of increase in fluorescence intensity with decreasing oxygen in the solution was measured and calculated as the change per minute. Each experiment was performed in at least 10 wells using different worms.

Basal and maximal OCR were measured using the Seahorse XFe24 Extracellular Flux analyzer (Agilent Technologies). Synchronized worms of D7 adulthood were washed three times with M9 buffer and transferred into M9‐filled wells in replicates four per condition. To generate stable OCR measurements, five measurement cycles were performed for basal OCR, nine cycles for maximal OCR following the addition of FCCP (10 μM final concentration), and four cycles for non‐mitochondrial OCR following the addition of sodium azide (10 mM final concentration). Basal OCR was determined as OCR before adding FCCP minus OCR after adding sodium azide. Maximal OCR was determined as OCR after adding FCCP minus OCR after adding sodium azide.

### 2.7 RNAi treatment

For *mcu‐1* RNAi, clones from the Ahringer RNAi feeding library (Source BioScience, Nottingham, UK) were used. The clone number was K02B2.3. RNAi was performed by bacterial feeding as described by Kamath et al.^26^ L4 larvae from the WT (*ccIs4251*; *aceIs1*), WT (*goeIs3*; *aceIs1*), and *dys‐1(eg33)* were transferred to NGM plates coated with HT115(DE3) bacteria expressing dsRNA for *mcu‐1* gene. Bacteria containing the empty L4440 vector were used as a control. The worms were transferred to newly prepared RNAi plates every 2 days.

### 2.8 Real‐time quantitative RT‐PCR

Total RNA was extracted from 25 to 30 synchronized worms with TRI reagent (Molecular Research Center, Cincinnati, OH, USA). cDNA was synthesized using a PrimeScript RT Reagent Kit with gDNA Eraser (Takara Bio Inc., Shiga, Japan). Real‐time quantitative RT‐PCR was performed with the following primer sets: *mcu‐1*: fw 5′‐CAC AAC AAC AGC CTC CTC AA‐3′, rv 5′‐GGC AAG GCT CAT TTC TTG AC‐3′; *hsp‐6*: fw 5′‐CAA ACT CCT GTG TCA GTA TCA TGG AAG G‐3′, rv 5′‐GCT GGC TTT GAC AAT CTT GTA TGG AAC G‐3′; *hsp‐60*: fw 5′‐AGG AAG AAA CGT GAT CAT CGA‐3′, rv 5′‐CAG CCT CCT CAT TAG CCT TG‐3′; *hsp‐4*: fw 5′‐GGT GTC GAA AAT ACC GGA GA‐3′, rv 5′‐ACT ATC GGC AGC GGT AGA GA‐3′; and *act‐1*: fw 5′‐CCT CTC CAC CTT CCA ACA GA‐3′, rv 5′‐AGA AAG CTG GTG GTG ACG AT‐3′. Data were normalized against the expression of *act‐1*. The experiment was repeated three times from different plates.

### 2.9 Measurement of mitochondrial ROS in body wall muscle cells

Mitochondrial ROS of body wall muscle cells was measured by MitoTracker™ Red CM‐H_2_Xros (M7513, Thermo Fisher Scientific, Waltham, MA, USA). The staining reagent at a final concentration of 10 μM was mixed with heat‐inactivated OP50 and spread onto the NGM plates to dry. N2 wild‐type and *mcu‐1(ju1154)* mutant worms were transferred and incubated with MitoTracker™ Red CM‐H_2_Xros for 2 days. Rotenone treatment was performed for 2 h prior to observation. The Images were detected by an FV10i confocal laser‐scanning microscope.

### 2.10 Ru360 treatment

Ru360 (Merck, Darmstadt, Germany) was dissolved in water and used at a final concentration of 10 μM. Eggs were laid on a medium containing Ru360 and grown.

### 2.11 Measurement of pH in body wall muscle cells

Intracellular pH of body wall muscle cells was measured by Invitrogen™ pHrodo™ Red AM Intracellular pH indicator (P35372, Thermo Fisher Scientific). Wild‐type N2 and untreated and Ru360‐treated *dys‐1(eg33)‐*mutant worms were incubated with 5 μM pHrodo™ Red AM Intracellular pH Indicator for 30 min at room temperature. Images were detected by an FV10i confocal laser‐scanning microscope. The pH was determined by a standard curve using Intracellular pH Calibration Buffer Kit (P35379, Thermo Fisher Scientific).

### 2.12 Statistical analysis

GraphPad Prism 9 software was used to determine statistical significance (GraphPad Software, San Diego, CA, USA). Statistical analyses were performed using the Student *t* test, one‐way ANOVA with Dunn's multiple comparison test, or the chi‐square test.

## 3 RESULTS

### 3.1 Age‐related mitochondrial changes with Ca^2+^ accumulation

**FIGURE 1:**
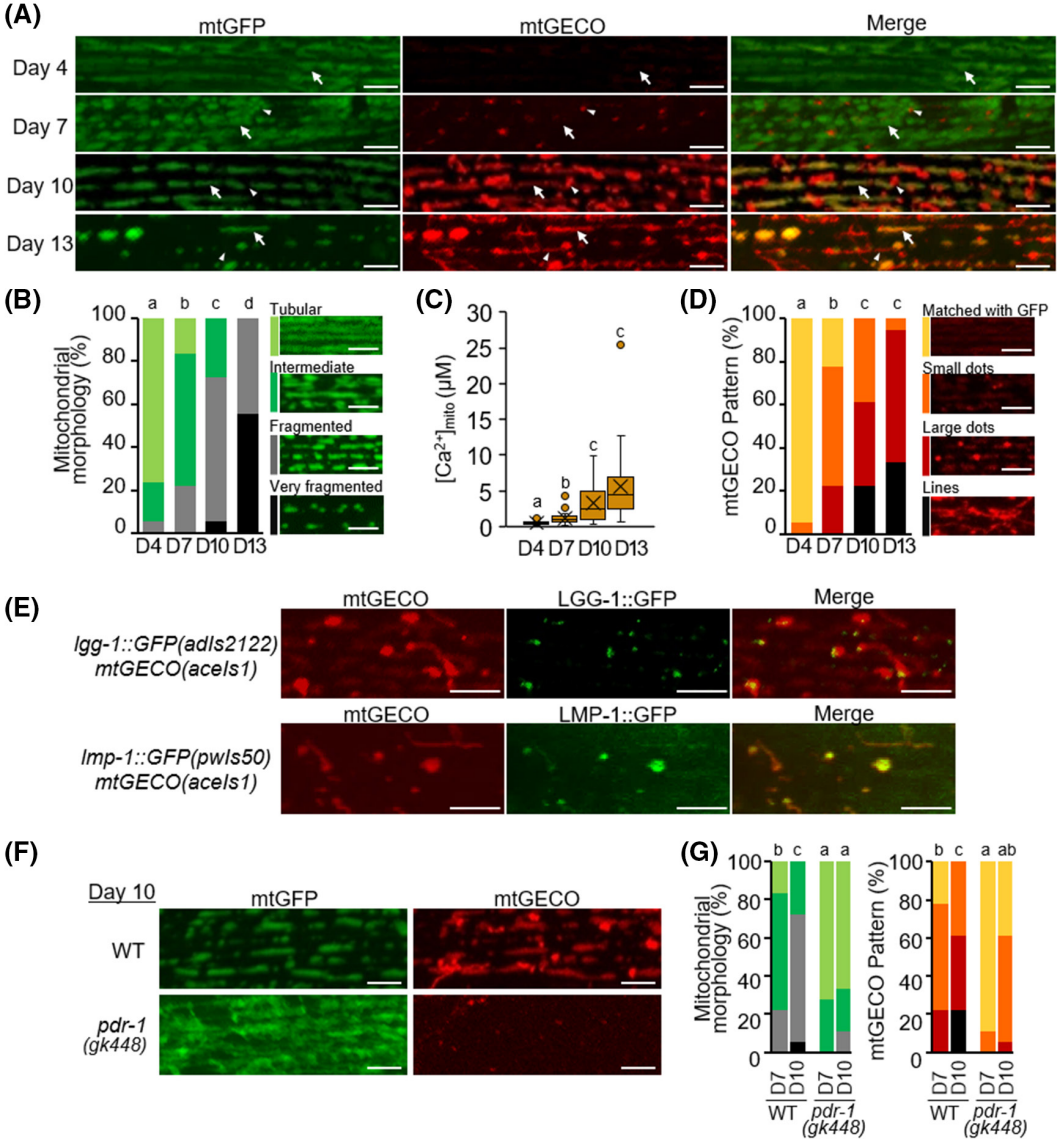
Decrease in mitochondrial volume and increase in mitochondrial Ca^2+^ level with age. (A) Representative fluorescence images of body wall muscle cells in transgenic *C. elegans* expressing mitochondria‐targeted GFP (mtGFP) and a mitochondrial Ca^2+^ probe (mtGECO) in the body wall muscle cells in WT worms (*ccIs4251; aceIs1*) at D4, D7, D10, and D13 of adulthood. The arrows indicate the typical position, where Ca^2+^ concentration in mitochondria ([Ca^2+^]_mito_) was measured in (C). The arrowheads indicate the position of mtGECO structures that are no longer colocalized with mtGFP. Scale bars: 5 μm. (B) Qualitative analysis of body wall muscle cells with abnormal mitochondrial morphology. On the left, light green, green, light gray, and black represent fractions containing cells with “tubular,” “intermediate,” “fragmented,” and “very fragmented” mitochondria, respectively. The right panels show representative images of the categorized mitochondria. Scale bars: 5 μm. Data represent the value from 18 cells from at least 6 worms. (C) Age‐dependent elevation in [Ca^2+^]_mito_ of muscle cells. Calcium levels of mtGFP‐positive mitochondria as shown by arrows in (A) were calculated from the ratio of fluorescence intensity of mtGECO to that of GFP in a constant area described in Materials and Methods. Data represent the value from 19 to 21 cells from at least six worms. (D) Qualitative analysis of body wall muscle cells with different mtGECO patterns. On the left, yellow, orange, red, and black represent fractions containing cells with the mtGECO signal “matched with GFP,” and the fractions of the cells that showed “small dots,” “large dots,” and “lines” of mtGECO signal, respectively. The right panels show representative images of the categorized patterns. Scale bars: 5 μm. Data represent the value from 18 cells from at least 6 worms. (E) Colocalization of mitophagy‐related proteins with mtGECO‐accumulated dots and tubular structures in D10 muscle cells. Representative fluorescence images of transgenic p*C. elegans* (*adIs2122* [*Plgg‐1*::*lgg‐1*::*GFP*]; *aceIs1*) expressing mtGECO and GFP‐tagged LGG‐1 and transgenic *C. elegans* (*pwIs50* [*Plmp‐1*::*lmp‐1*::*GFP*]; *aceIs1*) expressing mtGECO and GFP‐tagged LMP‐1. Scale bars, 5 μm. (F) Representative fluorescence images of mtGFP and mtGECO in body wall muscle cells in WT (*ccIs4251; aceIs1*) and *pdr‐1*(*gk448*) worms at D10 of adulthood. Scale bars: 5 μm. (G) Qualitative analysis of body wall muscle cells with abnormal mitochondrial morphology and different mtGECO patterns (*n* = 18 muscle cells from at least six worms). Color‐coding bars are the same as in (B) and (D). Letters on the tops of bars indicate statistical significance by the chi‐square test (B, D, G) or one‐way ANOVA with Dunn's multiple comparison tests (C) (*p* < .05).

In the body wall muscle in D4 adulthood (4 days after L4 stage) of WT worms, the mitochondria contained aligned filamentous structures, but these structures fragmented and shortened with increasing age (Figure 1A,B). Our qualitative analysis showed that more than 70% of muscular cells in the D10 WT worms were classified into “fragmented” or “very fragmented.” Next, we examined whether resting mitochondrial Ca^2+^ levels were elevated in the muscle cells of aged nematodes as previously observed in rodent models.^4^ To estimate mitochondrial Ca^2+^ concentrations, we used genetically modified WT worms that have mitochondria‐targeted red fluorescent Ca^2+^ indicator (mito‐LAR‐GECO1.2 (mtGECO))^24^ (*aceIs1* transgene; see Figure S2A) and mitochondrial‐ and nuclear‐targeted GFP (mtGFP and nucGFP) in their muscle cells (*ccIs4251* transgene).^21^ We imaged fluorescent signals of mtGECO in mtGFP‐positive structures and calculated the mtGECO/mtGFP ratio. The mtGECO/mtGFP ratio was converted to Ca^2+^ concentration from the obtained formula (see MATERIALS AND METHODS section). To determine whether artificial changes in the mtGECO/mtGFP ratio occur with aging, muscle mitochondria of each age were exposed by cutting of worms, and the fluorescent intensity of mtGECO and mtGFP was measured in the presence of Ca^2+^ and divalent cation ionophore ionomycin (Figure S1). The mtGFP‐positive structures in muscle cells of aged worms (D10) had about the same mtGECO/mtGFP values as D1 (Figure S1A,B). However, in an intact state (e.g., in vivo), mtGECO fluorescence on the mtGFP‐positive structures of aged worms was observed to be more intense and the mtGECO/mtGFP values were found to be larger in aged worms (Figures 1A and S1C,D). Since the *Kd* of mtGECO (LAR‐GECO1.2) is relatively high (12 μM),^24^ there were thought to be age‐dependent micromolar changes in mitochondrial Ca^2+^ concentration ([Ca^2+^]_mito_). Our quantitative analysis of [Ca^2+^]_mito_ estimated an increase with age from 0.6 ± 0.3 μM (D4) to 3.4 ± 2.8 μM (D10), and 5.6 ± 4.6 μM (D13) (Figure 1A,C), consistent with prediction. This is also consistent with results from a previous report using rodents, where mitochondrial Ca^2+^ levels increased with age.^4^

In *C. elegans*, age‐related mitochondrial fragmentation and disappearance have previously been reported.^14^, ^15^, ^16^ What is new in our study is that we found increased intramitochondrial Ca^2+^ levels (Figure 1A,C). Interestingly, we also found a greater extent in the mtGECO structures (“small dots,” “large dots,” and “lines”) that no longer colocalized with mtGFP (Figure 1A,D). The high‐intensity dots of mtGECO frequently appeared adjacent or connected to mtGFP structures. We suspected that the high‐intensity dots of mtGECO were Ca^2+^‐accumulated portions of mitochondria that would be eliminated by mitophagy to clear damaged mitochondria with age. This may be caused by the RFP fluorescence of mtGECO persisting, while the mitochondrial GFP fluorescence quenched. Indeed, 20 min of live imaging confirmed that the mtGECO‐only structures were isolated from the mitochondrial network, where both mtGFP and mtGECO were positive (Movie S1). In addition, the mtGECO structures colocalized with the autophagosomal marker LGG‐1::GFP, or with the lysosomal marker LMP‐1::GFP in D10 muscle cells (Figure 1E). To investigate the effects of mitophagy inhibition, we generated a Parkin homolog *pdr‐1(gk448)* mutant with mtGFP and mtGECO transgenes. The result showed that inhibition of mitophagy suppressed age‐related mitochondrial fragmentation and the formation of Ca^2+^‐accumulated mtGECO structures (Figure 1F,G). Taken together, these results suggest that the fragmented mitochondrial network in aged *C. elegans* muscle cells contains fragments with elevated Ca^2+^ levels and that these Ca^2+^‐accumulated portions of mitochondria are eliminated by mitophagic pathways, presumably to maintain mitochondrial function.

### 3.2 Effect of loss‐of‐function mutation in *mcu‐1* on muscle aging in *C. elegans*

**FIGURE 2:**
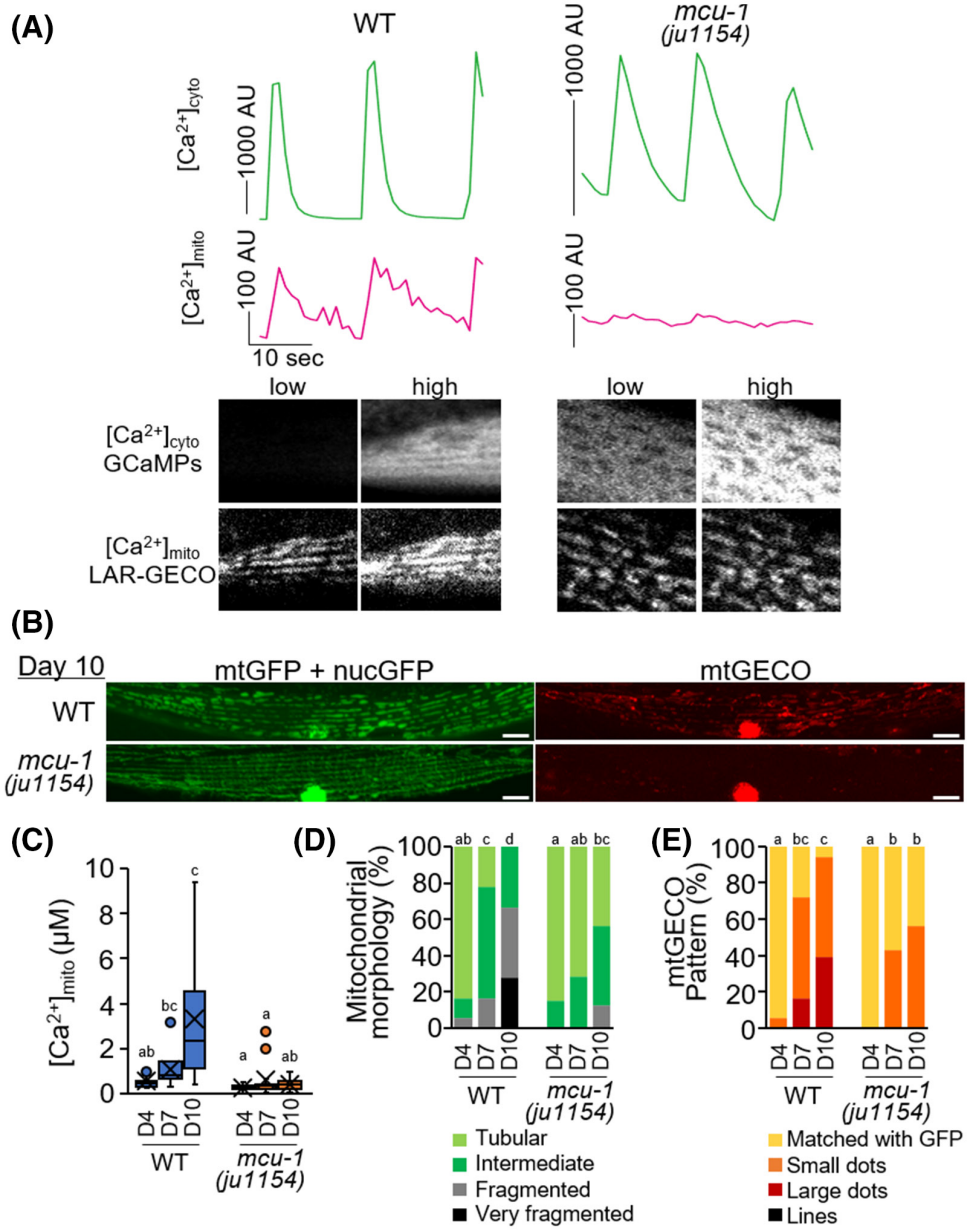
Effect of loss‐of‐function mutation in *mcu‐1* on muscle aging in *C. elegans*. (A) Fluorescent signals of cytosolic GCaMPs ([Ca^2+^]_cyto_, green) and mtGECO ([Ca^2+^]_mito_, magenta) in body wall muscles of immobilized WT worm (*goeIs3; aceIs1*, left panel) or *mcu‐1(ju1154)* mutant (right panel). Typical fluorescent images of muscle cytosolic GCaMP and mtGECO at low and high [Ca^2+^]_cyto_ levels were shown in the bottom panel. Mitochondrial Ca^2+^ uptake during cytosolic Ca^2+^ fluctuations was suppressed in *mcu‐1* mutants. (B) Representative fluorescence images of mitochondria‐targeted GFP (mtGFP), nuclear‐targeted GFP (nucGFP), and mitochondrial Ca^2+^ probe (mtGECO) in body wall muscle cells in WT (*ccIs4251; aceIs1*) and *mcu‐1(ju1154)* worms at D10 of adulthood. Mitochondrial fragmentation and mtGECO accumulation were abolished by *mcu‐1* mutation. Fluorescence signals on the nuclei observed in the “mtGECO” channel are from spectral bleed‐through from nucGFP (See also Figure S2B). Scale bars: 10 μm. (C) Loss‐of‐function mutation in *mcu‐1* decreased mitochondrial Ca^2+^ levels. Mitochondrial Ca^2+^ levels on mtGFP‐positive mitochondria in muscle cells on D4, D7, and D10 of WT or *mcu‐1(ju1154)* adulthood worms were calculated as described in Materials and Methods (*n* = 14). (D) Qualitative analysis of body wall muscle cells with abnormal mitochondrial morphology (*n* = 18–20 muscle cells from at least six worms). (E) Qualitative analysis of body wall muscle cells with different mtGECO patterns (*n* = 18–20 muscle cells from at least six worms). Letters on the tops of bars indicate statistical significance by one‐way ANOVA with Dunn's multiple comparison test (C), or the chi‐square test (D and E) (*p* < .05).

ATU2301 (WT) adult *C. elegans* expressing the genetically encoded Ca^2+^ indicator GCaMP in muscle cytosol (*goeIs3*)^22^ and mitochondrial Ca^2+^ indicator mtGECO in muscle mitochondria (*aceIs1*) were immobilized on polystyrene microspheres and observed live over time. Live imaging of cytosolic Ca^2+^ ([Ca^2+^]_cyto_) with contraction and relaxation of the body wall muscles was performed on D4, D10, and D15 of ATU2301. Immobilized *C. elegans* showed two typical patterns (single and continuous) of [Ca^2+^]_cyto_ transients in body wall muscle cells (Figure S3). A single Ca^2+^ transient peak is thought to be associated with a train of action potential spikes and muscle contraction.^27^ The result showed that the [Ca^2+^]_cyto_ peak width increased and height decreased with age (Figure S3). The full width half maximum (FWHM)^28^ was 5.8 ± 2.6 s, 15.0 ± 16.9 s, and 64.2 ± 35.9 s in D4, D10, and D15 of adulthood, respectively. Furthermore, in these D4 worms, cytosolic and mitochondrial Ca^2+^ levels fluctuated in synchronization with contraction and relaxation of the body wall muscles (Figure 2A and Movie S2). When a null mutation in the mitochondrial calcium uniporter *mcu‐1*(*ju1154*) was introduced into this recombinant (ATU2302; *mcu‐1*(*ju1154*)), [Ca^2+^]_cyto_ changes associated with muscle contraction were observed, but synchronized Ca^2+^ influx into the mitochondria was largely lost (Figure 2A and Movie S2). These results indicate that when a large amount of Ca^2+^ flows into the muscle cytosol due to muscle contraction, Ca^2+^ is also taken up in mitochondria via MCU‐1. Gibhardt et al. report similar usefulness of LAR‐GECO as a mitochondrial Ca^2+^ sensor in the mammalian muscular system.^29^ Intriguingly, the *mcu‐1* mutant was shown to suppress mitochondrial fragmentation and severe loss of mitochondrial mass, as well as age‐related Ca^2+^ accumulation in mitochondria (Figure 2B–D). Similarly, these suppressions were observed in the animals treated with *mcu‐1* RNAi (Figure S4). As a result, the proportion of muscle cells displaying progressing mitophagy with age also decreased (Figures 2B,E and  S4B,E).

**FIGURE 3:**
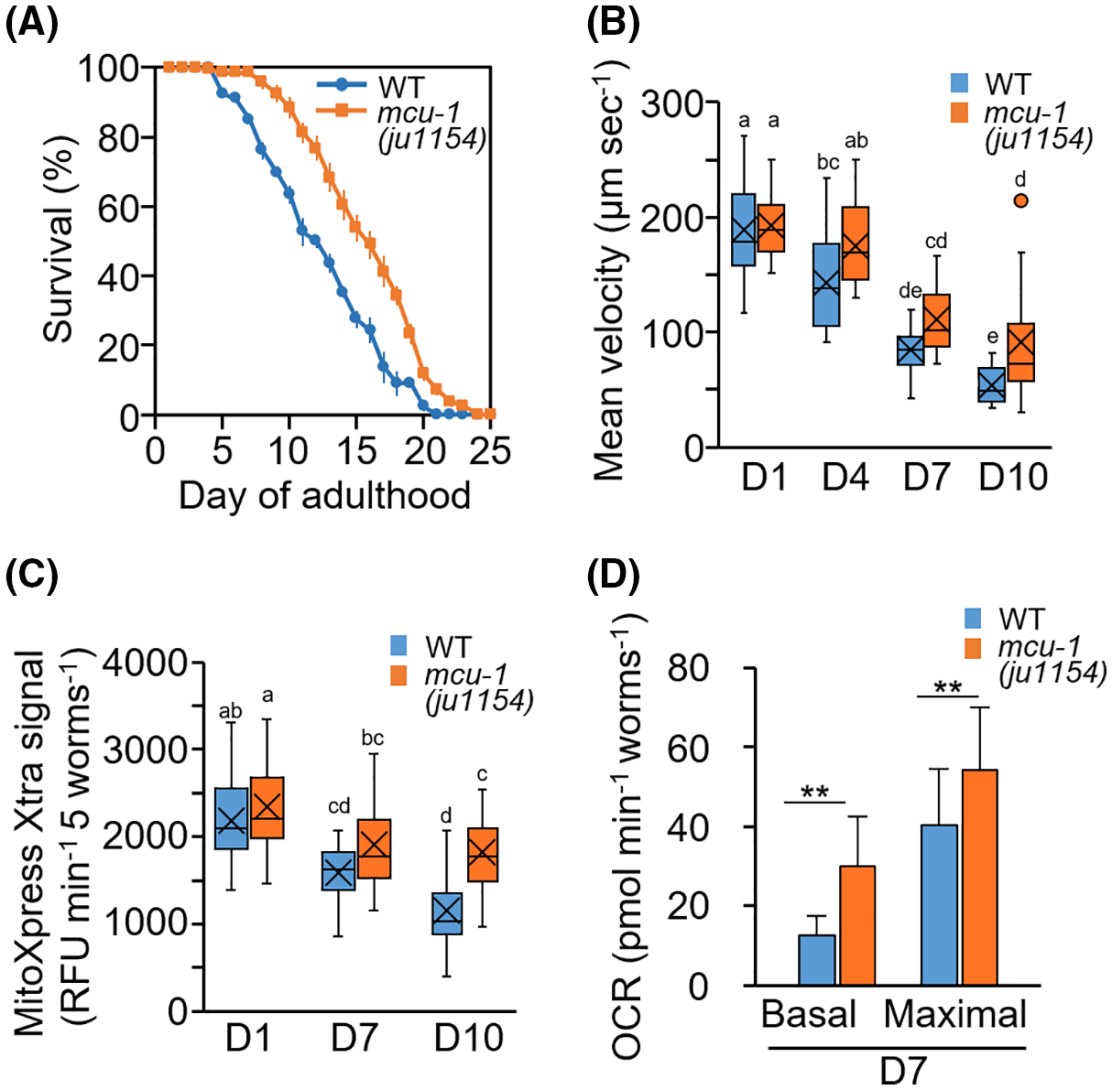
Life span, motor activity, and oxygen consumption rate in *mcu‐1* mutants. (A) Survival curve of WT (*ccIs4251; aceIs1*) and *mcu‐1(ju1154)* worms. Data are presented as the mean ± standard error from four plates with 25–30 worms per plate. (B) Mean velocity of WT and *mcu‐1(ju1154)* worms (*n* = 16–22 worms). Loss‐of‐function mutation in *mcu‐1* ameliorated age‐related reductions in movement velocity. (C) The respiration rate of WT and *mcu‐1(ju1154)* mutant worms. The respiration rate was measured using a MitoXpress Xtra oxygen consumption assay kit and expressed as the relative fluorescence unit (RFU) per minute per five worms (Materials and Methods). The age‐related reduction was improved in the mutants. Data represent the value from at least 10 repeats, each with five worms. (D) Basal and maximal OCR levels at D7 of WT and *mcu‐1(ju1154)* at D7 of adulthood. Data represent the value from four repeats, each with at least six worms. Letters on the tops of bars indicate statistical significance by one‐way ANOVA with Dunn's multiple comparison tests (B and C) or Student's *t* test (D) (**p < .01).

In the control wild‐type (WT) with *aceIs1* and *ccIs4251* transgenes, approximately half of the population had died by 10‐day‐old adulthood (D10). In the *mcu‐1* mutant, the life span was extended; the difference in life expectancy between the mutant and control was 3.2 days (control, 12.5 ± 0.2 days; *mcu‐1(ju1154)*, 15.7 ± 0.4 days; *p* < .01) (Figure 3A). A similar result was observed between wild‐type N2 and the mutant strain CZ19982 without the transgenic reporters; the original *mcu‐1(ju1154)* mutation extended life span (N2, 14.7 ± 0.5 days; CZ19982 *mcu‐1(ju1154)*, 17.6 ± 0.8 days; *p* < .05). Furthermore, *mcu‐1* null mutant maintained a higher motor activity (mean velocity) than WT (Figure 3B). The respiration rate was measured using a MitoXpress Xtra oxygen consumption assay kit (Figure 3C). The age‐related decline was significantly reduced in the *mcu‐1(ju1154)* null mutation in the same background. A similar tendency was observed in the basal and maximal oxygen consumption rate (OCR) of D7 adults measured by the Seahorse XFe24 Extracellular Flux analyzer (Figure 3D). Activation of the mitochondrial unfolding protein response (UPR^mt^) has been reported to promote longevity and also to protect against environmental stresses.^30^, ^31^ Therefore, it is possible that in *mcu‐1* mutants UPR^mt^ might be activated to prevent age‐related mitochondrial fragmentation. However, in *mcu‐1* mutants, the expression levels of *hsp‐6* and *hsp‐60*, which are responsible for UPR^mt^,^32^, ^33^ were unexpectedly decreased (Figure S5). These results suggest that the reduction of UPR^mt^ activity in *mcu‐1* mutants is due to the maintenance of mitochondrial homeostasis. Mitochondrial Ca^2+^ has also been reported to increase mitochondrial ROS levels,^34^ and *mcu‐1* mutants might improve age‐related mitochondrial fragmentation by decreasing ROS levels. However, no reduction in mitochondrial ROS levels was detected in *mcu‐1* mutants (Figure S5).

### 3.3 Effect of pharmacological inhibition of MCU‐1with Ru360 on muscle aging in *C. elegans*

**FIGURE 4:**
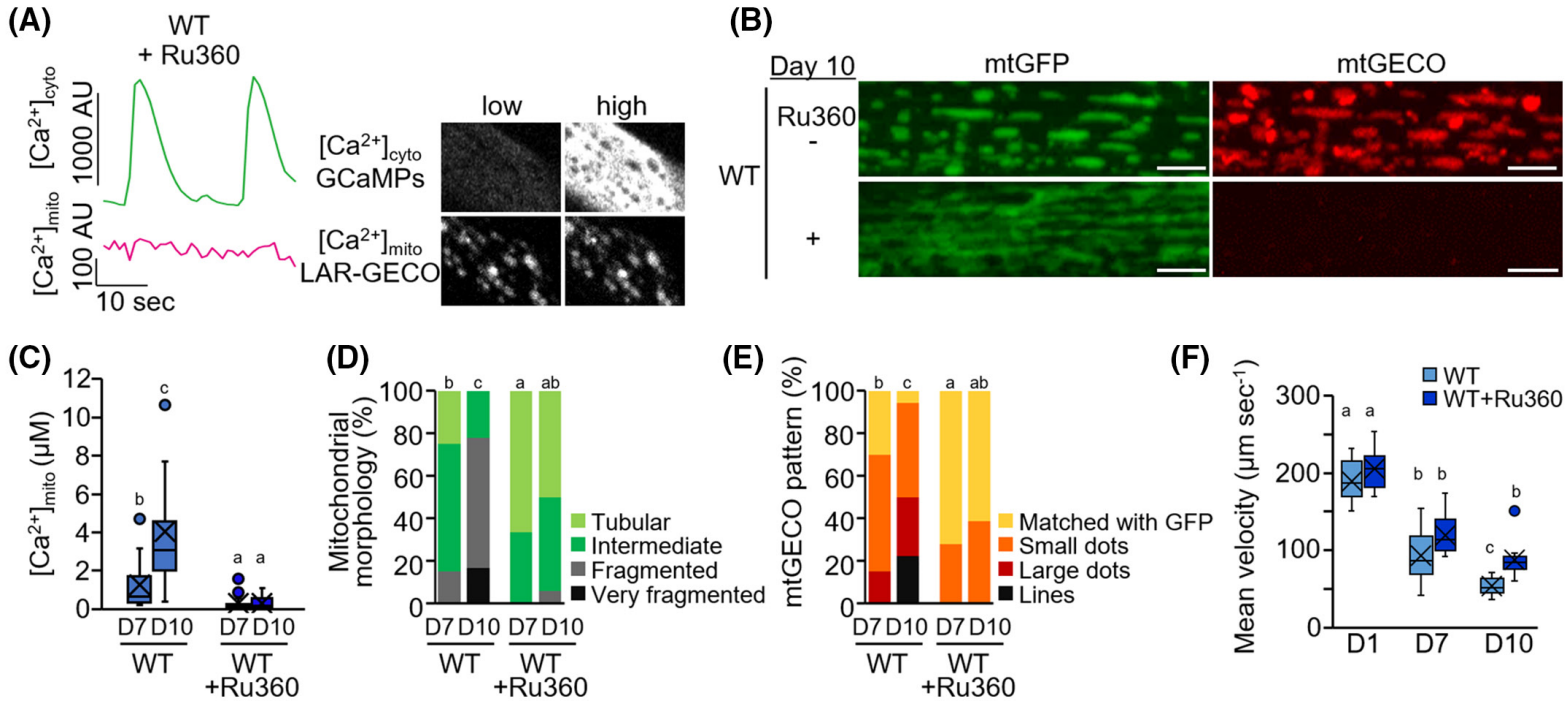
Pharmacological inhibition of *mcu‐1* with Ru360 on muscle aging in *C. elegans*. (A) Suppression of mitochondrial Ca^2+^ uptake during cytosolic Ca^2+^ fluctuations by Ru360 treatment. Fluorescent signals of cytosolic GCaMPs ([Ca^2+^]_cyto_, green) and mtGECO ([Ca^2+^]_mito_, magenta) were monitored simultaneously in body wall muscles of Ru360‐treated WT worm (*goeIs3; aceIs1*). Typical fluorescent images of muscle cytosolic GCaMP and mtGECO at low and high [Ca^2+^]_cyto_ levels were shown in the right panel. (B) Representative fluorescent images of mitochondria (mtGFP) and a mitochondrial Ca^2+^ probe (mtGECO) in body wall muscle cells in untreated and Ru360‐treated WT worms (*ccIs4251; aceIs1*) at D10 of adulthood. Ru360 treatment suppressed age‐related mitochondrial fragmentation and mtGECO accumulation. Scale bars: 5 μm. (C) Mitochondrial Ca^2+^ levels in mtGFP‐positive mitochondria of muscle cells on D7 and D10 of adulthood. The levels were calculated as described in Materials and Methods (*n* = 14). Ru360 treatment restrained the age‐related increase in mitochondrial Ca^2+^ levels. (D) Qualitative analysis of body wall muscle cells with abnormal mitochondrial morphology (*n* = 18 muscle cells from at least six worms). (E) Qualitative analysis of body wall muscle cells with different mtGECO patterns (*n* = 18 muscle cells from at least six worms). (F) Mean velocity of untreated and Ru360‐treated WT worms (*ccIs4251; aceIs1*) (*n* = 10–13 worms). Ru360 treatment improved the age‐related decline in mean velocity. Letters on the tops of bars indicate statistical significance by one‐way ANOVA with Dunn's multiple comparison test (C and F) or the chi‐square test (D and E) (*p* < .05).

Having established that genetic ablations of *mcu‐1* were sufficient to improve age‐related muscle mitochondrial changes, we examined whether pharmacological inhibition of MCU‐1 could similarly improve muscle health with age. Ru360, a specific mitochondrial calcium uptake inhibitor,^35^, ^36^ was used to inhibit mitochondrial Ca^2+^ influx. As this compound is significantly restricted in intact mammalian systems due to its poor cell permeability,^35^, ^36^ we first evaluated the penetration of Ru360 into intact *C. elegans* continuously cultured at a final concentration of 10 μM Ru360 from egg to adulthood. In adults of ATU2301 treated with Ru360, synchronized Ca^2+^ influx into the mitochondria was significantly lost (Figure 4A and Movie S3), as it was the *mcu‐1* null mutant (Figure 2A and Movie S2), indicating that Ru360 is permeable to *C. elegans* muscle cells. As expected, Ru360 treatment prevented age‐related changes in the body wall muscles of *C. elegans* including upregulated [Ca^2+^]_mito_, mitochondrial fragmentation, and formation of mtGECO structures (Figure 4B–E). Furthermore, Ru360 improved mobility with age (Figure 4F). These results confirm that inhibition of MCU‐1‐mediated Ca^2+^ influx attenuates age‐dependent changes in mitochondrial morphology and Ca^2+^ levels, suppresses the emergence of Ca^2+^‐accumulated structures, and ultimately attenuates movement decline with age.

### 3.4 Improvement of health by inhibition of *mcu‐1* in the *C. elegans* DMD model

**FIGURE 5:**
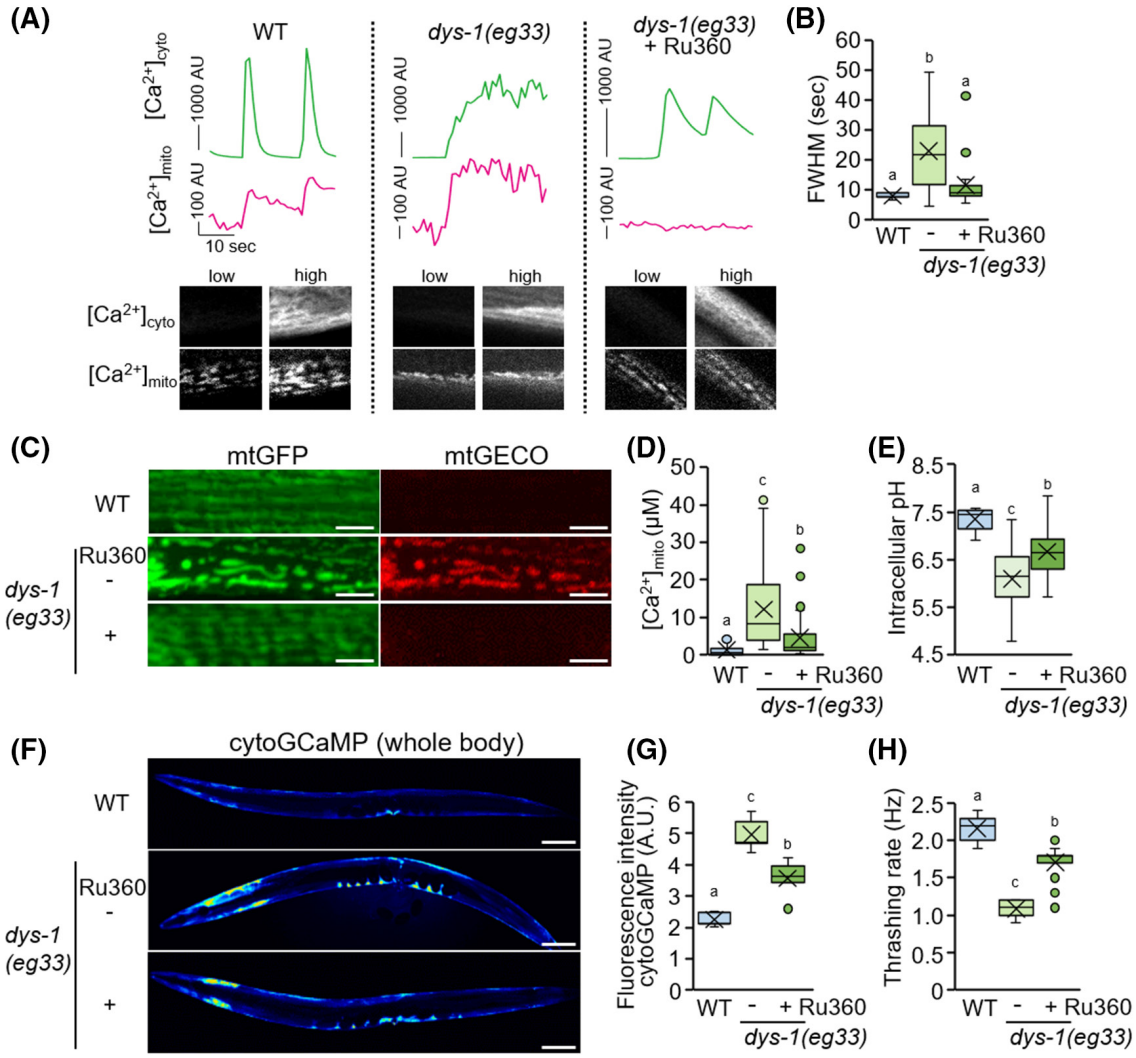
Effect of Ru360 treatment on *C. elegans* dystrophy model. (A) Mitochondrial Ca^2+^ uptake (magenta) during spontaneous cytosolic Ca^2+^ fluctuations (green) in body wall muscles of immobilized WT worm (*goeIs3; aceIs1*, left panel), *dys‐1(eg33)* (center panel), and Ru360‐treated *dys‐1(eg33)* (right panel). Fluorescent signals of cytosolic GCaMPs and mtGECO were monitored simultaneously. Typical fluorescent images of muscle cytosolic GCaMP and mtGECO at low and high levels were shown in the bottom panel. (B) Full width half maximum (FWHM) of the [Ca^2+^]_cyto_ peaks in WT (*goeIs3; aceIs1*), untreated and Ru360‐treated *dys‐1(eg33)* mutant worms (*n* = 16–20). (C) Representative fluorescent images of mitochondria and mtGECO in the body wall muscle cells. Scale bars: 5 μm. (D) Mitochondrial Ca^2+^ levels in mtGFP‐positive mitochondria of muscle cells on D2 of adulthood. The levels were calculated as described in Materials and Methods (*n* = 31–39). (E) Intracellular pH in body wall muscle cells in WT (N2) and untreated and Ru360‐treated *dys‐1(eg33)* mutant worms on D3 of adulthood (*n* = 11–13). (F and G) Fluorescent images (F) and fluorescence intensity (G) of the whole body of transgenic *C. elegans* expressing a cytosolic Ca^2+^ probe (GCaMP) in body wall muscle cells. The *dys‐1(eg33)* mutant exhibited higher fluorescence intensity for cytosolic GCaMP than the WT worms, and Ru360 treatment rescued this change. Scale bars: 100 μm. (H) Thrashing rate (Hz) in WT (*goeIs3; aceIs1*), untreated and Ru360‐treated *dys‐1(eg33)* mutant worms. Inhibition of mitochondrial Ca^2+^ uptake by Ru360 improved the decline in motor activity (thrashing) of the *dys‐1(eg33)* mutant (*n* = 25). Letters on the tops of bars indicate statistical significance by one‐way ANOVA with Dunn's multiple comparison test (B, D, E, G, H) (*p* < .05).

In the *C. elegans* DMD model *dys‐1(eg33)*, altered calcium homeostasis in muscle is a primary pathology. These dystrophin mutants display increased mitochondrial fragmentation in muscle cells and decreased mobility.^17^, ^18^ Given the positive effects of inhibition of *mcu‐1* on muscle calcium homeostasis and health with age, we were curious if Ru360 could have beneficial effects on the dystrophy model. After immobilization with polystyrene microspheres, [Ca^2+^]_cyto_ live imaging was performed on single muscle cells of D4 animals (Figure 5A and Movie S4). The FWHM duration of the *dys‐1*(*eg33*) mutant, was significantly (23.0 ± 13.5 s) longer than that of the wild type (8.0 ± 0.8 s), suggesting that muscle rigidity occurred (Figure 5A,B). FWHM broadened in *dys‐1(eg33)* D4 animals and was similar to WT‐aged animals. In addition, [Ca^2+^]_mito_ also maintained higher levels in mutant muscle cells (Figure 5A). Intriguingly, Ru360 treatment not only suppressed Ca^2+^ influx into mitochondria but also significantly improved the broadening of FWHM (11.6 ± 7.7 s) in the *dys‐1* mutants (Figure 5A,B). We also found that fragmentation of mitochondria and Ca^2+^ accumulation in mitochondria in the muscle cells of dystrophin‐mutant worms were decreased by Ru360 treatment (Figure 5C,D).

In both mammalian cells and *C. elegans* body wall muscle cells, intracellular acidification is caused by mitochondrial fragmentation and the pH drops from 7.5 to about 7.0.^37^ Although measured with different pH indicators, we obtained similar results with wild‐type pH 7.4 versus pH 6.1 in the *dys‐1* mutant and pH 6.7 in Ru360 treated *dys‐1* mutants (Figure 5E). In addition, we monitored Ca^2+^ level in the cytosol of the muscle cells on D2 of adulthood after anesthesia with sodium azide. Compared with the WT counterparts, the dystrophin mutants had significantly higher [Ca^2+^]_cyto_ levels, and surprisingly, Ru360 treatment decreased [Ca^2+^]_cyto_ levels in dystrophin mutants (Figure 5F,G). Furthermore, Ru360 improved mobility in the *dys‐1* mutants (Figure 5H).

**FIGURE 6:**
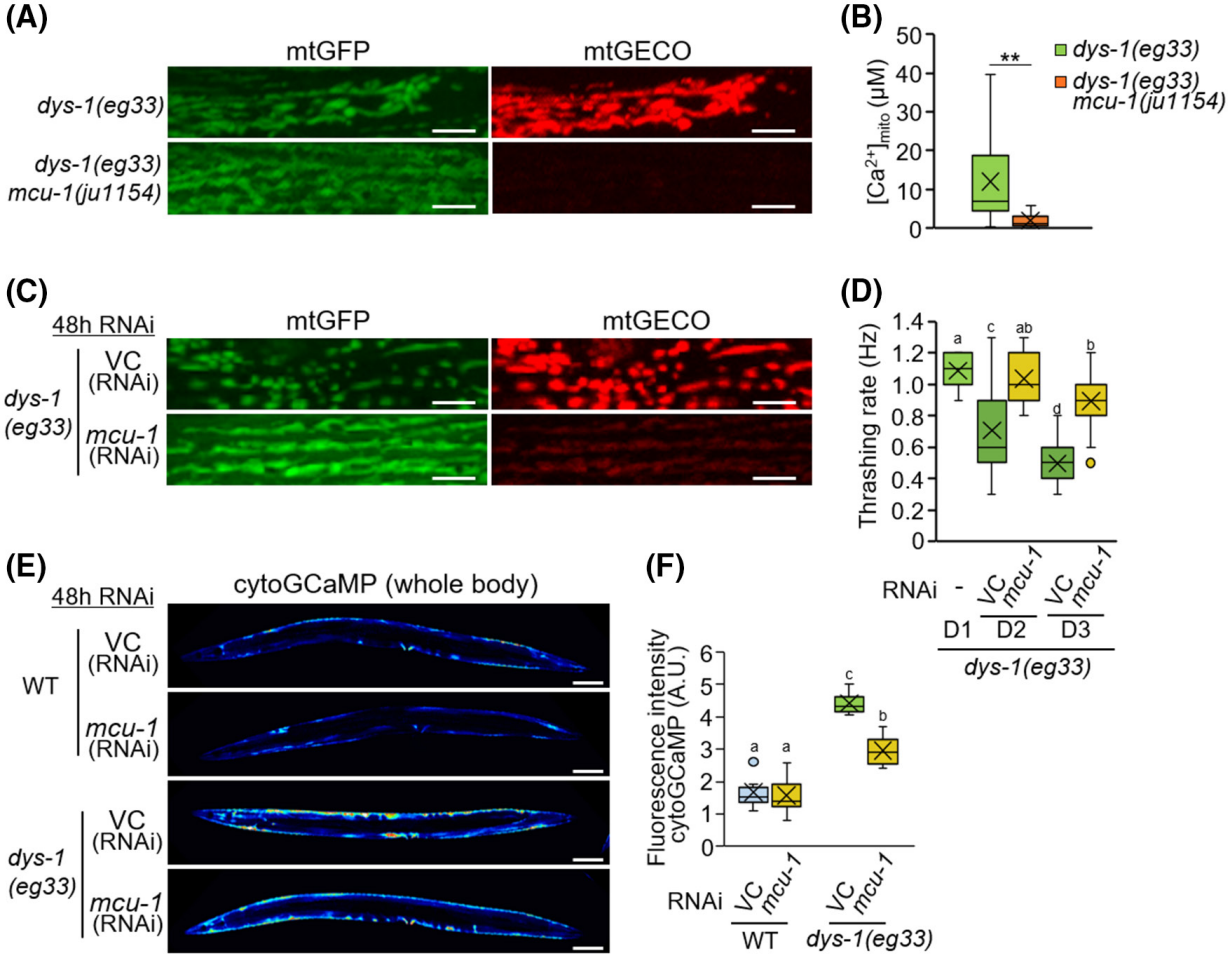
Improvement of dystrophy model by inhibition of *mcu‐1* in *C. elegans*. (A) Representative fluorescent images of mtGFP and mtGECO in the body wall muscle cells in *dys‐1(eg33)* and *dys‐1(eg33)*;*mcu‐1(ju1154)* double‐mutant worms at D2 of adulthood. Mitochondrial fragmentation and mtGECO accumulation were abolished by *mcu‐1* null mutation. Scale bars: 5 μm. (B) Mitochondrial Ca^2+^ levels in mtGFP‐positive mitochondria of muscle cells on D2 of adulthood. The levels were calculated as described in Materials and Methods (*n* = 30). (C) Representative fluorescence images of mtGFP and mtGECO in the body wall muscle cells in the *dys‐1(eg33)* mutant after 48 h of RNAi treatment (VC: vector control, and *mcu‐1*). Scale bars: 5 μm. (D) Thrashing rate (Hz) in RNAi treated *dys‐1(eg33)* mutant worms. Inhibition of mitochondrial Ca^2+^ uptake by *mcu‐1* RNAi improved the decline in motor activity (thrashing) of the *dys‐1(eg33)* mutant (*n* = 25). (E and F) Fluorescent images (E) and fluorescence intensity (F) of the whole body of transgenic *C. elegans* expressing a cytosolic Ca^2+^ probe (GCaMP) in body wall muscle cells. The *dys‐1(eg33)* mutant exhibited higher fluorescence intensity for cytosolic GCaMP than the WT worms, and *mcu‐1* RNAi rescued this change. Scale bars: 100 μm. Letters on the tops of bars indicate statistical significance by one‐way ANOVA with Dunn's multiple comparison tests (B, D, F) (*p* < .05).

Similar to Ru360 treatment, *mcu‐1(ju1154)* null mutation was shown to suppress mitochondrial fragmentation and Ca^2+^ accumulation in mitochondria in the muscle cells of dystrophin‐mutant worms (Figure 6A,B). RNAi of *mcu‐1* prevented the progression of mitochondrial Ca^2+^ accumulation and decline in the movement of dystrophin‐mutant worms (Figure 6C,D). The level of [Ca^2+^]_cyto_ was decreased in dystrophin mutants by *mcu‐1* RNAi (Figure 6E,F). These results indicate that suppression of mitochondrial Ca^2+^ influx improves muscular Ca^2+^ homeostasis in the *dys‐1*(*eg33*) mutant and restores the motility dysfunction. Thus, elevated mitochondrial Ca^2+^ causes impaired muscle health not only with age but also in DMD.

## 4 DISCUSSION

With society rapidly aging, overcoming the frailty that accompanies extended life expectancy is a major issue. Sarcopenia, which is age‐related muscular atrophy, a 2% annual loss of skeletal muscle in healthy people over the age of 60, is directly related to frailty and mortality.^1^, ^2^, ^3^ During this progression, muscle mitochondrial alterations such as reduced volume, irregular morphology, and decreased functional activity are also observed in aged mice and older people.^4^, ^5^, ^6^ An experimental model, the nematode *C. elegans*, is advantageous for aging studies because it has a relatively short life span and is basically similar to vertebrate systems at the molecular level. Similar decreases in mitochondrial volume and increased fragmentation occur earlier than disruption of muscle sarcomere structures and correlate more strongly with decreased maximal velocity and life span.^14^, ^15^, ^16^ In this study, using the mitochondrial Ca^2+^ sensor mtGECO,^24^ we showed that *C. elegans* age‐related mitochondrial fragmentation and loss are caused by mitophagic removal of Ca^2+^ accumulated portions of mitochondria (Figure 1). Thus, inhibition of the function of the mitochondrial Ca^2+^ uniporter MCU (either genetic ablation of *mcu‐1* or pharmacological inhibition with Ru360) prevented the age‐related elimination of impaired mitochondrial portions by mitophagy (Figures 2 and 4). It led to the mitigation of age‐related mitochondrial volume loss and mobility disability (Figures 2, 3, 4). We also performed a rescue experiment of *mcu‐1(ju1154)* under the body wall muscle promoter because *mcu‐1* is widely expressed in other tissues. However, we attempted to transform *mcu‐1* mutants using the construct *Pmyo3::mcu‐1*, but these recombinants lost motility and were unable to develop into adults. This suggests that high expression of *mcu‐1* is lethal. Rescue experiments with lower expression of *mcu‐1* require further analysis.

The mtGFP‐negative mtGECO structures were trafficked into acidic lysosomal compartments, which must have quenched the fluorescence of GFP. In a weakly acidic environment, mtGECO can fluoresce but decrease fluorescence emission as the pH decreases.^24^ The high fluorescence of mtGECO in lysosomes, despite a decrease in fluorescence emission in acidic environments, suggests that the Ca^2+^ concentration may be high in the lysosome, but further analysis is needed using a Ca^2+^ sensor that does not change fluorescence value under acidic conditions. The expression pattern of mtGFP‐negative mtGECO structures was matched with the LMP‐1 (Figure 1E). In *C. elegans* body wall muscle cells, LMP‐1 is known to be localized to both tubular and round‐shaped vesicles.^38^ It is also known that autophagy target proteins that colocalized with LMP‐1, such as misfolded proteins and aggregated proteins, are not only degraded in one cell but are also transported to neighboring cells and released from muscle cells into the intestine and coelomocytes.^38^ Therefore, the Ca^2+^‐accumulated portion of mitochondria is incorporated into lysosomes by mitophagy and might be exported from muscle cells by cell‐to‐cell transport.

In mammals, it is known that mitochondrial fragmentation and muscle dysfunction are associated with elevated levels of mitochondrial ROS.^39^ In *C. elegans*, increased mitochondrial ROS by rotenone treatment leads to mitochondria fragmentation.^40^ However, in this study, age‐related mitochondrial fragmentation in body wall muscle cells could be rescued by inhibiting mitochondrial Ca^2+^ accumulation (Figures 2, 4, S4), although inhibition hardly changed mitochondrial ROS levels (Figure 5A,B). These results indicate that age‐related muscle mitochondrial fragmentation and volume loss are primarily driven by mitophagy activation via mitochondrial Ca^2+^ accumulation in *C. elegans*. Also, reducing Ca^2+^ accumulation in age‐related mitochondria leads to maintain healthy mitochondria (Figure 3).

Ca^2+^ is essential for optimal mitochondrial function, but its overload impairs mitochondrial function, leading to decreased mitochondrial inner membrane potential (ΔΨm) and ATP production, increased ROS release, and ultimately cell death.^41^ Moreover, since mitochondria can uptake large amounts of [Ca^2+^]_cyto_, this sequestration alters the quantitative and dynamic properties of Ca^2+^ signaling in both cytosol and mitochondria.^42^, ^43^ In *C. elegans* body wall muscle cells, cytosolic and mitochondrial Ca^2+^ levels fluctuated synchronously with contraction and relaxation (Figure 2A and Movie S2). Our results also indicate that Ca^2+^ is also taken up into mitochondria via MCU‐1 when muscle contraction triggers a large Ca^2+^ influx into the cytosol (Figures 2A and 4A, Movies S2 and S3). Recently, it has been reported that the highly conserved ryanodine receptor (RyR), UNC‐68 in *C. elegans*, is oxidized with age which results in age‐dependent “leaky” channels.^44^ Live imaging of [Ca^2+^]_cyto_ with contraction and relaxation of the body wall muscles showed that the [Ca^2+^]_cyto_ peak width significantly increased with age (Figure S3). These observations suggest that age‐related RyR dysfunction causing prolonged elevation of [Ca^2+^]_cyto_ may contribute to the increase in muscle [Ca^2+^]_mito_ with age.

DMD, the most severe and common muscular dystrophy that early mimics age‐related muscular atrophy, is a severe progressive muscle disease caused by mutations in the gene encoding dystrophin. Similar to human DMD, the *C. elegans dys‐1(eg33)* mutation synthesizes a C‐terminal truncated dystrophin protein that loses scaffolding function.^45^ In *dys‐1(eg33)* mutants, similar to prednisone and H_2_S donor treatment (NaGYY),^17^, ^46^ *mcu‐1* inhibition ameliorated the severely fragmented mitochondrial network and restored motility (Figure 5C,H). Furthermore, *mcu‐1* inhibition significantly suppressed the increase in [Ca^2+^]_cyto_ levels in *dys‐1* mutants (Figures 5F,G and 6E,F). On the other hand, the loss of [Ca^2+^]_cyto_ homeostasis is not improved by either prednisone or H_2_S supplementation.^17^ Therefore, controlling MCU function is likely to work through a different mechanism than H_2_S supplementation and prednisone. Interestingly, pharmacological activation of Sarco/endoplasmic reticulum Ca^2+^‐ATPase (SERCA) was recently reported to ameliorate the dystrophic phenotypes in *mdx* mice.^48^ Administration of SERCA activation reduced [Ca^2+^]_cyto_ levels, reversed mitochondrial swelling, increased OCR, and decreased ROS production in isolated mitochondria of *mdx* mice in vitro and ex vivo. Thus, pharmacological activation of SERCA may suppress mitochondrial Ca^2+^ overaccumulation and ameliorate the muscular dystrophic phenotype.

Our present study shows that MCU inhibition (genetic ablation of *mcu‐1* and Ru360 treatment) ameliorated muscular function decline with age and DMD in *C. elegans*. In contrast, in MCU knockout mice, skeletal muscle showed altered phosphorylation and activity of pyruvate dehydrogenase, significantly impairing the ability to perform strenuous work.^48^ Rizzuto's group also shows that MCU silencing causes muscle atrophy.^10^ On the other hand, recently, MCU‐1 inhibitors, which are also ruthenium compounds with improved in vivo permeability, such as Ruthenium Red and Ru265, have been developed and are being investigated as potential therapeutic agents for cardiac dysfunctions.^49^, ^50^ These findings suggest MCU has an evolutionarily conserved role in muscle health. Further, differences in the role of MCU function with development and growth vs. aging and pathology are now apparent. Likewise, mutations in an MCU regulator MICU1, which increases resting mitochondrial Ca^2+^ levels, caused neuromuscular disorders with cognitive decline, muscle weakness, and an extrapyramidal motor disorder.^11^ In particular, the aging state in mammalian muscles, where muscle satellite cells are gradually lost and the regenerative capacity is reduced,^51^ is highly similar to the aging of body wall muscle cells in adult *C. elegans*. Together suggest that controlling MCU function can be the potential target for diagnosis of sarcopenia even in the mammalian system.

## 5 CONCLUSIONS

Here we show that the blockage of MCU‐1function by genetic or pharmacological modulation improves health in both aging *C. elegans* and *C. elegans* with muscular dystrophy. These observations suggest that loss of calcium homeostasis is an early event in muscle aging that can be mitigated by improving mitochondrial calcium buffering capacity. Suppression of mitochondrial Ca^2+^ influx prevented the formation of Ca^2+^‐accumulated structures in body wall muscle cells. These age‐associated Ca^2+^‐accumulations appear to normally be removed via mitophagy. These results suggest that RyR, which causes increased Ca^2+^ with age, MCU‐1, which facilitates an increase mitochondrial Ca^2+^ with age, and mitophagy, which maintains mitochondrial homeostasis, are part of a coordinated system that fails to maintain muscle health with age and which may be targeted for improved muscle health.

## AUTHOR CONTRIBUTIONS

Atsushi Higashitani and Takeshi Kobayashi conceived and designed the study. Mika Teranishi, Yui Nakagawa, Yukou Itoh, Surabhi Sudevan, Takeshi Kobayashi, and Atsushi Higashitani conducted experiments and analyzed the data. Yukihiko Kubota contributed to the generation of transgenic *C. elegans*. Atsushi Higashitani, Mika Teranishi, Surabhi Sudevan, Nathaniel J Szewczyk, and Takeshi Kobayashi wrote the manuscript. Nathaniel J. Szewczyk and Takaaki Abe supervised the mitochondrial dysfunction project and chemical treatment. All authors read and approved the final paper.

## DISCLOSURES

The authors declare that they have no competing interests.

## Supporting information


Video S1:

Video S2:

Video S3:

Video S4:

Figure S1‐S4

## Data Availability

All experimental data of this study are available from the authors upon reasonable request. The ATU2301, ATU3301, and ATU4301 are available from the Caenorhabditis Genetic Center (CGC). The other ATU series of nematode strains constructed in this study are available from the authors in accordance with the Material Transfer Agreement.
