# Supplementary material for: Increased mitochondrial Ca2+ contributes to health decline with age and Duchene muscular dystrophy in C. elegans
Source: FASEB J. 2023 Mar 19;37(4):e22851. doi: 10.1096/fj.202201489RR (PMC10946577; doi:10.1096/fj.202201489RR)
Supplement: Supplementary file 5 — Figure S1‐S4 [file FSB2-37-e22851-s002.pdf]

Supplementary Figures

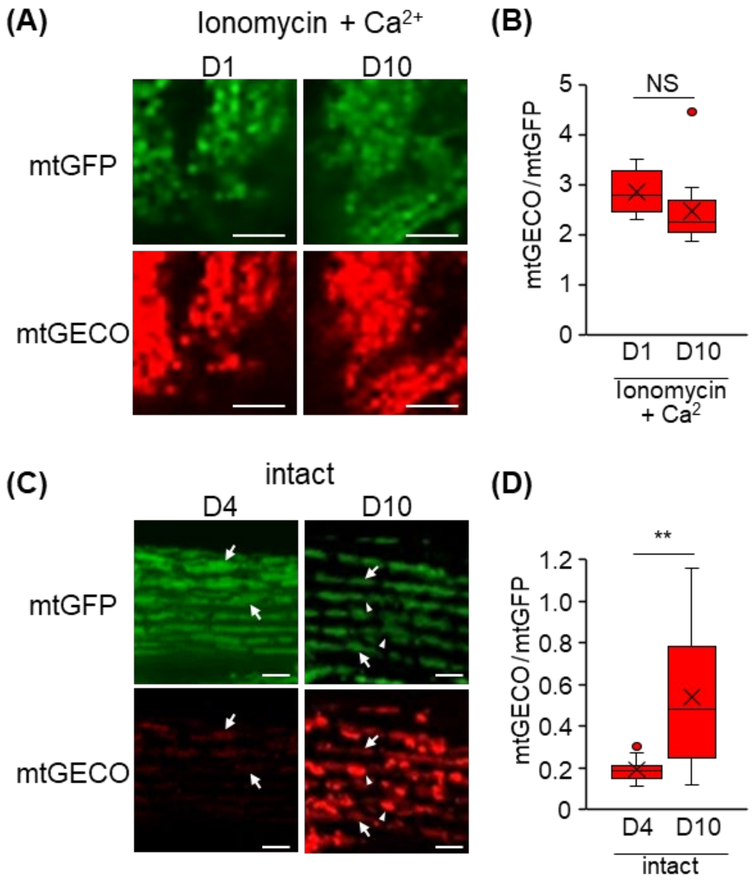

Figure S1. Measurement of mitochondrial Ca^{2+} levels in body wall muscle cells

(A) Representative fluorescence images of mtGFP and mtGECO in the mitochondria of body wall muscle cells in WT worms (*ccIs4251; aceIs1*). Muscle mitochondria at D1 and D10 of adulthood were exposed by the incision of worms with a blade and then mixed with Ionomycin in $CaCl_2$. Scale bars, 5 µm. (B) The ratio of fluorescence intensity of mtGECO to mtGFP with Ionomycin in $CaCl_2$. No significant difference was observed between D1 and D10. NS means not significant ($p > 0.05$, Student's *t*-test) (n=6-13). (C) Representative fluorescence images of mtGFP and mtGECO in the intact body wall muscle cells in WT worms (*ccIs4251; aceIs1*). The arrows indicate the typical position where the ratio of mtGECO to mtGFP was detected. The arrowheads indicate the position of mtGECO structures that no longer colocalized with mtGFP. Scale bars, 5 µm. (D) The ratio of fluorescence intensity of mtGECO to mtGFP in the intact body wall muscle cells. The value was significantly different between D4 and D10 (** $p < 0.01$, *t*-test) (n=19-21).

(A)

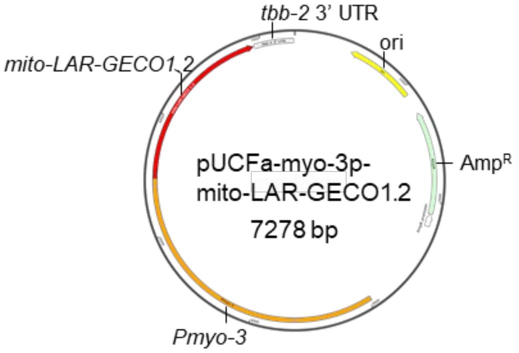

(B)

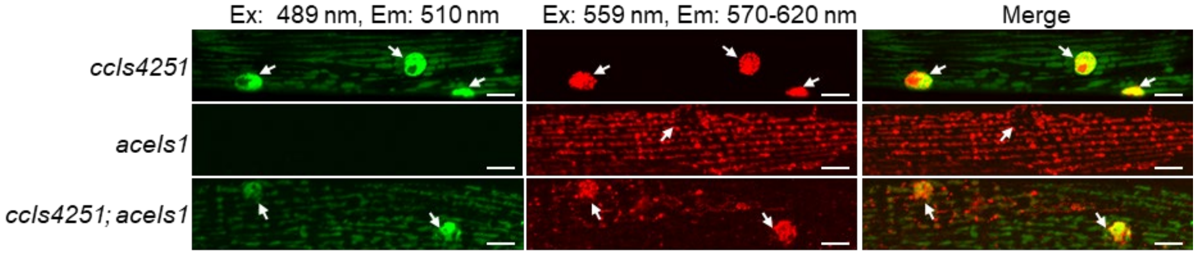

Figure S2. The construct of mitochondrial-targeted LAR-GECO1.2 in *aceIs1* and fluorescence of *ccIs4251* in body wall muscle cells.

(A) Plasmid map of the construct used to introduce the *aceIs1* transgene. Mitochondrial-targeted LAR-GECO1.2 was constructed under the *myo-3* promoter with the *tbb-2* 3' UTR. The LAR-GECO 1.2 codon was optimized for expression in *C. elegans*. pUC19 was used as a vector backbone. (B) The *ccIs4251* transgene showed not only GFP but also red fluorescence (not merged with GFP) in the muscle nuclei. *C. elegans* strain SD1347 (*ccIs4251* [*Pmyo-3*::*GFP-LacZ(NLS)* + *Pmyo-3*::*mitochondrial GFP*]), ATU4301 (*aceIs1* [*Pmyo-3::mitochondrial LAR-GECO* + *Pmyo2::RFP*]), and ATU3301 (*ccIs4251;aceIs1*) were monitored. The *ccIs4251* transgene shows red fluorescence in the muscle nuclei under the detection conditions for mtGECO (excitation: 559 nm, emission: 570-620 nm: FV10i confocal laser-scanning microscope, Olympus). The arrows indicate the position of nuclei. Scale bars, 5 µm.

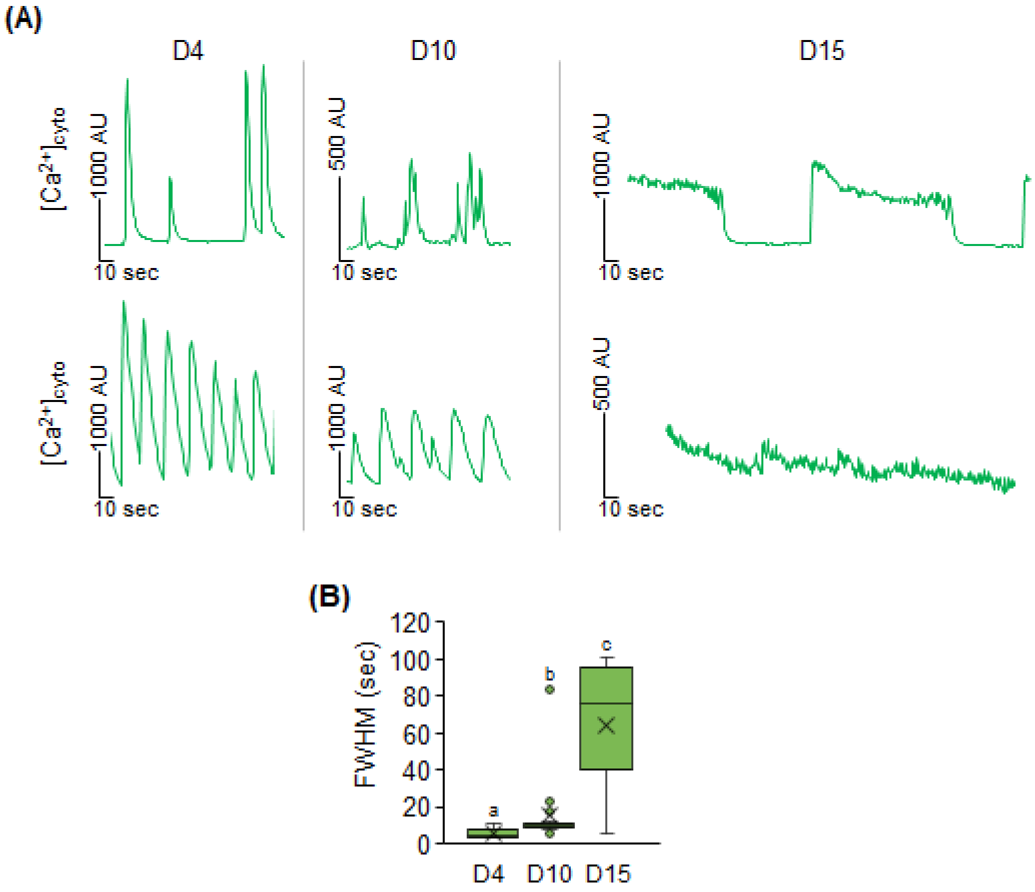

Figure S3. Age-related increase in cytosolic Ca^{2+} level in body wall muscle cells.

(A) Fluorescent signals of cytosolic GCaMPs ($[Ca^{2+}]_{cyto}$) in body wall muscles of immobilized WT worm (*goeIs3; aceIs1*) at D4, D10 and D15 of adulthood. Immobilized *C. elegans* show two typical patterns of $[Ca^{2+}]_{cyto}$ transients in body-wall muscle cells at each age: single and continuous patterns. (B) Full-width half-maximum (FWHM) of the $[Ca^{2+}]_{cyto}$ peaks in WT at D4, D10 and D15 of adulthood (n=10-18). FWHM increased with age. Letters on the tops of bars indicate statistical significance by one-way ANOVA with Dunn's multiple comparison test ($p < 0.05$).

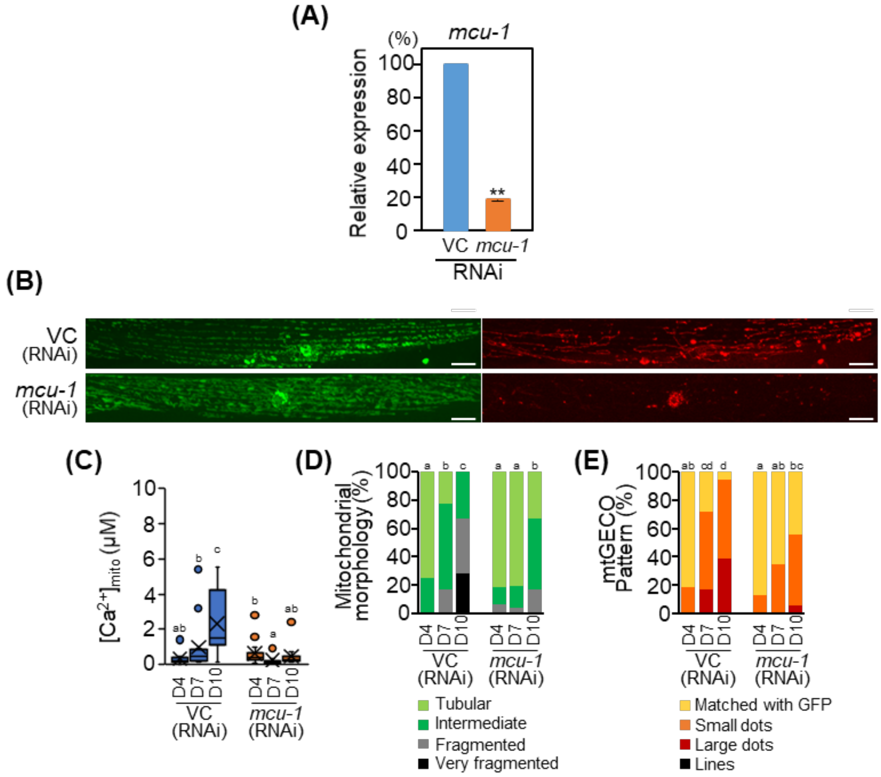

Figure S4. Effect of *mcu-1* RNAi on muscle aging in *C. elegans*

(A) The efficiency of RNAi treatment. WT worms (*ccIs4251; aceIs1*) were treated with a RNAi sequence (VC: vector control or *mcu-1*) from L4 larvae. Total RNA was extracted on D7 of adulthood, and RT-PCR was performed using sequence-specific primers. The data indicate a relative value with the value of VC set to 100%. Error bars represent standard errors among the four plates with 25-30 worms per plate (** $p < 0.01$, Student's *t*-test). (B) Representative fluorescence images of mitochondria-targeted GFP (mtGFP), nuclear-targeted GFP (nucGFP), and mitochondrial Ca^{2+} probe (mtGECO) in body wall muscle cells of RNAi treated WT worms (*ccIs4251; aceIs1*) (VC and *mcu-1*) on D10 of adulthood. Scale bars, 10 μm. (C) Mitochondrial Ca^{2+} levels on mtGFP-positive mitochondria in muscle cells on D4, D7 and D10 adulthood worms treated with RNAi sequence was calculated as described in Materials and Methods (n=14). (D) Qualitative analysis of body wall muscle cells with abnormal mitochondrial morphology (n=18-20 muscle cells from at least 6 worms). (E) Qualitative analysis of body wall muscle cells with different mtGECO pattern (n=18-20 muscle cells from at least 6 worms).

Letters on the tops of bars indicate statistical significance by one-way ANOVA with Dunn's multiple comparison test (C) or the chi-square test (D,E) ($p < 0.05$).

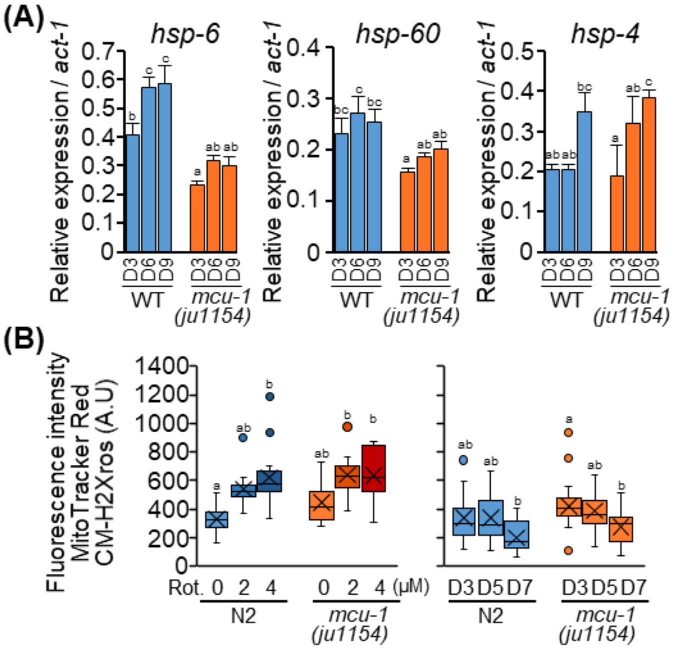

Figure S5. UPR^{mt} and mitochondrial ROS levels in *mcu-1* mutants

(A) Expression level of genes known to be induced by mitochondrial UPR^{mt} (*hsp-6* and *hsp-60*) and UPR^{er} (*hsp-4*). The *act-1* gene was used as the internal standard. Data are means of three biological repeats. Error bars indicate SD. (B) Fluorescence intensity of MitoTracker™ Red CM-H_2Xros (mtROS) in body wall muscle cells. WT worms (N2) and CZ19982 *mcu-1(ju1154)* at D3 of adulthood were treated with 0 (Mock), 2 and 4 μM Rotenone for 2h (n=12) (left). Fluorescence intensity of mtROS in N2 and CZ19982 *mcu-1(ju1154)* at D3, D5, and D7 of adulthood (n=15-20) (right).

Letters on the tops of bars indicate statistical significance by one-way ANOVA with Dunn's multiple comparison test (** $p < 0.01$).
